# Association between durable response (DR) and overall survival (OS) in patients with unresected stage IIIb-IV melanoma treated with Talimogene Laherparepvec (T-VEC) in the Phase III OPTiM trial

**DOI:** 10.1186/2051-1426-2-S3-P91

**Published:** 2014-11-06

**Authors:** Howard L Kaufman, Robert HI Andtbacka, Frances A Collichio, Michael Wolf, Ai Li, Mark Shilkrut, Igor Puzanov, Merrick Ross

**Affiliations:** 1 Rutgers Cancer Institute of New Jersey, New Brunswick, NJ, USA; 2 Huntsman Cancer Institute, Salt Lake City, UT, USA; 3 The University of Carolina at Chapel Hill, Chapel Hill, NC, USA; 4 Amgen Inc, Thousand Oaks, CA, USA; 5 Vanderbilt University Medical Center, Nashville, TN, USA; 6 MD Anderson Cancer Center, Houston, TX, USA

## Background

T-VEC is an HSV-1-derived oncolytic immunotherapy designed to selectively replicate within tumors, produce GM-CSF and enhance systemic antitumor immune responses. In OPTiM (NCT00769704), a randomized Phase III trial of intralesional T-VEC vs subcutaneous GM-CSF for unresected stage IIIB-IV melanoma, T-VEC significantly improved DR rate (partial response or complete response lasting continuously for ≥6 months; primary endpoint) vs GM-CSF (16% vs 2%, p < 0.0001). In the primary overall survival (OS) analysis of the ITT population, median OS was 4.4 months longer in the T-VEC arm than in the GM-CSF arm (23.3 months vs 18.9 months; HR = 0.79, 95%CI: 0.62-1.00; *P *= 0.051; Kaufman et al. ASCO 2014; abs9008a). Here we evaluate an association between DR and OS.

## Methods

To avoid lead-time bias, OS was compared for those patients who were alive and who achieved DR vs those who did not at landmark times of 9, 12 and 18 months from randomization. A Cox proportional hazards model with achievement of DR as a time-varying indicator was also evaluated. Potential bias due to confounding was evaluated with sensitivity analyses adjusting for prognostic factor imbalances.

## Results

The analysis included all patients in the T-VEC ITT arm (n = 295) who were alive at the 9 (n = 234), 12 (n = 209) and 18 (n = 165) month landmark times. At 9, 12 and 18 months, 20, 33 and 47 patients had a DR, and 214, 176 and 118 did not. For patients who had a DR vs those who did not, HRs for improved OS were 0.08 (95% CI: 0.01-0.56), 0.05 (95% CI: 0.01-0.39) and 0.13 (95% CI: 0.03-0.54) at 9, 12 and 18 months, respectively, indicating that achieving a DR is associated with improved OS. When DR was analyzed as a time-dependent covariate, the HR favoured patients achieving a DR (HR: 0.09; 95% CI: 0.03-0.29).

## Conclusion

Achieving a DR was associated with marked decrease in the risk of death (> 85% across various analyses). Although the design of the study prevents demonstrating a causal relationship between DR and prolonged OS, it is reasonable to assume that achieving a durable response would lead to improvement in OS, and these analyses support that assumption. DR should be further explored as a surrogate for OS in patients with melanoma treated by immunotherapy.

